# Coverage Extension and Balancing the Transmitted Power of the Moving Relay Node at LTE-A Cellular Network

**DOI:** 10.1155/2014/815720

**Published:** 2014-01-29

**Authors:** Jaafar A. Aldhaibani, Abid Yahya, R. Badlishah Ahmad

**Affiliations:** ^1^School of Computer and Communication Engineering, University Malaysia Perlis (UniMAP), Perlis, 01000 Kangar, Malaysia; ^2^Department of Space Technology and Communication, Ministry of Science and Technology, Baghdad, Iraq

## Abstract

The poor capacity at cell boundaries is not enough to meet the growing demand and stringent design which required high capacity and throughput irrespective of user's location in the cellular network. In this paper, we propose new schemes for an optimum fixed relay node (RN) placement in LTE-A cellular network to enhance throughput and coverage extension at cell edge region. The proposed approach mitigates interferences between all nodes and ensures optimum utilization with the optimization of transmitted power. Moreover, we proposed a new algorithm to balance the transmitted power of moving relay node (MR) over cell size and providing required SNR and throughput at the users inside vehicle along with reducing the transmitted power consumption by MR. The numerical analysis along with the simulation results indicates that an improvement in capacity for users is 40% increment at downlink transmission from cell capacity. Furthermore, the results revealed that there is saving nearly 75% from transmitted power in MR after using proposed balancing algorithm. ATDI simulator was used to verify the numerical results, which deals with real digital cartographic and standard formats for terrain.

## 1. Introduction

Long-term evolution-advanced (LTE-A) is the enhancing of the 3rd generation partnership project (3GPP) LTE, which improves LTE features in terms of coverage and throughput [1]. The relay is one of the major innovations of LTE-A, to meet growing demand for coverage extension, throughput, capacity enhancement, and saving the high deployment cost whether if deploying small size BS as solution to increase the coverage. The basic idea of relaying is that the relay received the signals from source and forwarded these signal after amplification to the destination node.

**Figure 1:**
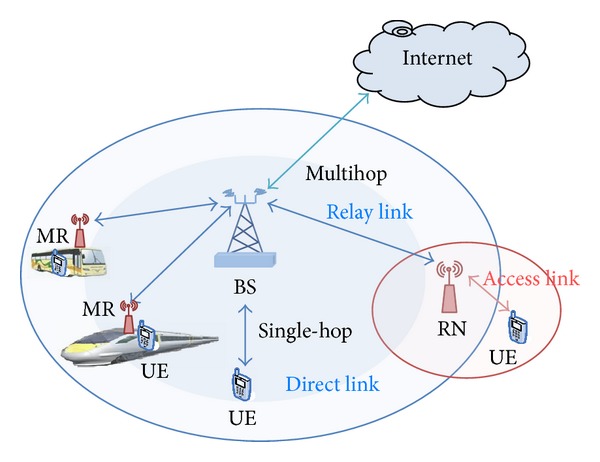
Relay nodes scenarios.

On relaying scenarios, there are two types of relaying architectures: fixed relay node (RN) and moving relay node (MR), where RNs are deployed near cell edge to increase the coverage and enhancing the throughput at the users in this region [2]. However, this improvement in coverage and throughput is based on the relay placement which provides fairness distribution of coverage within cell size as shown in Figure 1.

MR is the same kind of functionality as the RN but with the difference that they offer it while moving with the users. MR is new innovation to improve the throughput for vehicular users at LTE-A networks where it can be deployed flexibly to increase the throughput for passengers in buses or trains over rural area in cases where RNs are not available or not economically justifiable and the weak received signal from BSs [3].

MR is installed on vehicle and connected wirelessly with the BS via relay link (RL) and with passengers via access links (AL), so the MR and passenger are called group mobility [4] as shown in Figure 1. In fact, group mobility can be provided anywhere a large number of users are moving together during is using cellular network services.

The MR makes these services more reliable, with the assumption that the RL has a much better channel than regular UEs [5]. MR is connected to external power source via a battery charger or has its own power supply unit. This allows MRs to have a relatively high access to processing capabilities and to constant higher transmission powers.

Using MRN in cellular systems is still under discussion in the 3GPP LTE [6]. Studies have shown that through deploying symmetrical and cooperative relays on top of trains, the quality-of-service (QoS) of a UE inside the vehicle can be significantly improved.

The main contributions of this paper are deriving the optimal relay node by considering the saturation throughput distance near the nodes locations, which is estimated from 200 to 500 m according to stations design and antenna configuration. This distance yields more accurate results in order to provide maximum achievable rate to users and increasing the number of active users at cell edge region. Furthermore the second contribution in this paper is proposing the balancing power algorithm which is reduced the transmitted power of moving relay within vehicle along with enhancing throughput for passengers.

## 2. System Model Description

Half-duplex mode is proposed in this work, where the relay cannot transmit and receive simultaneously. In general, while UE moves away from the cell-center, SINR degrades due to two factors. Firstly, the received signal strength goes down as the path loss increases with distance from the BS. Secondly, the intercell-interference rises because UE moves away from one BS and approach another BS.

At the cellular network without relaying with assuming that UE is connected to BS_*i*_ and moving away towards BS_*j*_. The signal transmitted from BS_*j*_ appears as interference to the UE. The received signal at the downlink for each user *k* without relaying can be represented as the following equation:

(1) $$\begin{array}{c} Y_{i,k}=\sqrt{P_{i}}H_{i,k}X_{i,k}+\overset{N_{\text{cell}}}{\underset{j=0}{\sum }}\sqrt{P_{j}}H_{j,k}X_{j,k}+N_{k}, \end{array}$$

where *j* = 0 → *N*_cell_, *N*_cell_ is the number of neighboring cell; *P*_*i*_ and *P*_*j*_ are the transmit power of donor BS and neighboring BSs, respectively; *H*_*i*,*k*_ and *H*_*j*,*k*_ are the fading channel gain for donor and neighboring cell, respectively; and *N*_*k*_ is AWGN for user *k* [6, 7]:

(2) $$\begin{array}{c} \rho_{i,k}=\frac{P_{i}{\left|H_{i,k}\right|}^{2}}{N_{k}+\sum_{j=0}^{N_{\text{cell}}}P_{j}{\left|H_{j,k}\right|}^{2}}, \end{array}$$

where *ρ*_*i*,*k*_ is the SINR from the *i*th link in each single sub-carrier (*k*) UEs.

In a severely limited interference scenario, the background noise *N*_*k*_ can be ignored to simplify the calculations and (2) can be written as

(3) $$\begin{array}{c} \rho_{i,k}=\frac{P_{i}G_{r,k}G_{t,i}{\left(\lambda /4\pi \right)}^{2}D_{i,k}^{-\alpha }}{\sum_{j=1}^{N}P_{j}G_{r,k}G_{t,j}{\left(\lambda /4\pi \right)}^{2}d_{j,k}^{-\alpha }},\\ \rho_{i,k}=\frac{P_{i}{LD}_{i,k}^{-\alpha }}{\sum_{j=1}^{N}P_{j}L_{j}d_{j,k}^{-\alpha }}. \end{array}$$

The channel *H* is the function of path loss; therefore,

(4) $$\begin{array}{c} {\left|H\right|}^{2}={LD}^{-\alpha }, \end{array}$$

where *L* = *G*_*r*_ *G*_*t*_(*λ*/4*π*)^2^ is constant depending on the infrastructure of sender and receiver, *G*_*r*_, *G*_*t*_ is the antenna gains of the transmitter and receiver, respectively, *D*_*i*,*k*_ and *d*_*j*,*k*_ are the distances from user to donor BS_*i*_ and neighbour BS_*j*_, respectively, and *α* is the path loss exponent [8].

### 2.1. Capacity of Cell without Relay

Using adaptive modulation and coding AMC is one of the basic enabling techniques in the standards for 3G wireless networks that have been developed to achieve high spectral efficiency on fading channels [9]. Typically the quality of the signal received by a UE depends on channel quality from BS, level of interference from neighboring cells, and noise level. For a given modulation, the code rate can be chosen depending on the radio link conditions. At the downlink data transmissions in LTE-A, the BS usually selects the code of modulation scheme according to the channel quality indicator (CQI) feedback transmitted by the UE in the uplink [10].

**Figure 2:**
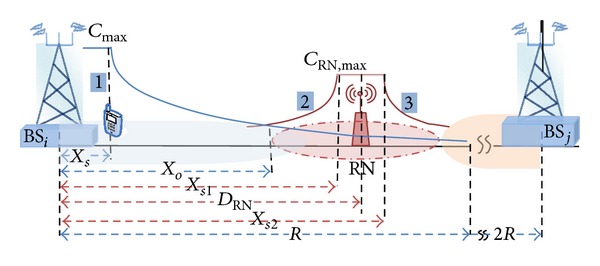
Scheme of proposed node locations.

This work splits the down link capacity from BS into two regions according to modulation and coding scheme (MCS) to provide realistic transmission schemes, as shown in Figure 2.

Based on [11, 12] the capacity in a single-input single-output LTE system can be estimated by

(5) $$\begin{array}{c} C_{i}=\min\left\{C_{\max},\text{B}{\text{W}}_{\text{eff}}\;\text{lo}{\text{g}}_{2}\left(1+\frac{\rho_{i}}{\rho_{\text{eff}}}\right)\right\}, \end{array}$$

where *C*_*i*_ is the estimated spectral efficiency in bps/Hz and *C*_max⁡_ is the upper limit based on the hard spectral efficiency given by 64-quadrature amplitude modulation with the coding rate of 0.753 equal to 4.32 bps/Hz [10]. *ρ*_*i*_ is the SINR for each user in the cell, BW_eff_ is the adjustment for the system bandwidth efficiency, and *ρ*_eff_ is the adjustment for the SINR implementation efficiency.

(BW_eff_, *ρ*_eff_) has the value of (0.56, 2.0) in the downlink and (0.52, 2.34) in the uplink [13]. The proposed system used two regions in cell capacity distribution. The first region around the BS is known as the saturation throughput region which is specified from 0 → *X*_*s*_, in which the level capacity is always steady based on the used modulation scheme, while the other region is determined from *X*_*s*_ → *R*, where in this region, the cell capacity is never steady based on the Shannon theory, as shown in Figure 2. According to these concepts the system performance can be described as the following equations. The received signal at UE in location (1) can be written as

(6) $$\begin{array}{c} Y_{i,X_{s}}=\sqrt{P_{i}}H_{i,X_{s}}X_{i,X_{s}}+\overset{N_{\text{cell}}}{\underset{j=0}{\sum }}\sqrt{P_{j}}H_{j,X_{s}}X_{j,X_{s}}+N_{X_{s}}. \end{array}$$

The ideal SINR at *X*_*s*_ location is

(7) $$\begin{array}{c} \rho_{\text{ideal}}=\frac{P_{i}LX_{s}^{-\alpha }}{P_{j}L_{1}{\left(2R-X_{s}\right)}^{-\alpha }}, \end{array}$$

where *L*_1_ is constant depending on the infrastructure of neighbor BSs. The error vector magnitude (EVM) is a measure of the difference between the ideal symbols and the measured symbols after the equalization [14]. This difference is called the error vector magnitude. For 64QAM modulation in LTE-A the SINR (*ρ*_*i*,*X*_*s*__) at *X*_*s*_ location is explained as [14, 15]

(8) $$\begin{array}{c} \frac{1}{\rho_{i,X_{s}}}=\frac{1}{1/\rho_{\max}+1/\rho_{\text{ideal}}}, \end{array}$$

where

(9) ρideal=PiLXs−αPjL1(2R−Xs)−α,ρi,Xs=ρidealρmax⁡ρideal+ρmax⁡=ρmax⁡PiLXs−α/PjL1(2R−Xs)ρmax⁡+PiLXs−α/PjL1(2R−Xs)−α.

Through simple mathematical processes we get

(10) $$\begin{array}{c} X_{s}=\frac{2R}{1+{\left(P_{i}L/P_{j}L_{1}\right)}^{-1/\alpha }{\left(\rho_{i,X_{s}}\right)}^{1/\alpha }-{\left(\rho_{\max}\right)}^{1/\alpha }}. \end{array}$$

For downlink LTE network the *ρ*_max⁡_ = 0.08 with 64QAM [14, 16]. The distance *X*_*s*_ depends on the infrastructure of the sender and the effect of interference from other cells. In the baseline outdoor capacity analysis, this work considers a three-sector omnidirectional antenna, with each sector having 120-degree diversity to avoid the interference and applying frequency reuse scheme. If all BSs have the same characteristics in LTE networks, then the constant region distance from the BS is

(11) $$\begin{array}{c} X_{s}=\frac{2R}{1+{\left(\rho_{i,X_{s}}\right)}^{1/\alpha }-{\left(\rho_{\max}\right)}^{1/\alpha }}, \end{array}$$

(12) $$\begin{array}{c} C_{X_{s}}={\text{BW}}_{\text{eff}}\;\text{lo}{\text{g}}_{2}\left(1+\frac{\rho_{i,X_{s}}}{\rho_{\text{eff}}}\right). \end{array}$$

From (5), logically, the total capacity over cell is equal or more than the *C*_max⁡_ capacity:

(13) $$\begin{array}{c} \text{B}{\text{W}}_{\text{eff}}\;\text{lo}{\text{g}}_{2}\left(1+\frac{\rho_{i,X_{s}}}{\rho_{\text{eff}}}\right)\geq C_{\max},\\ \rho_{i,X_{s}}\geq \rho_{\text{eff}}\left(2^{\left(C_{\max}/\text{B}{\text{W}}_{\text{eff}}\right)}-1\right). \end{array}$$

By substation in (11),

(14) $$\begin{array}{c} X_{s}\leq \frac{2R}{1+{\left(\rho_{\text{eff}}\left(2^{\left(C_{\max}/\text{B}{\text{W}}_{\text{eff}}\right)}-1\right)\right)}^{1/\alpha }-{\left(\rho_{\max}\right)}^{1/\alpha }}. \end{array}$$

According to (14), it is easy to evaluate the capacity saturation distance, for example, *X*_*s*_ equals 313.5 m if *α* = 2.2, *C*_max⁡_ = 4.32 bps/Hz, *ρ*_max⁡_ = 0.08, and 2500 m radius.

### 2.2. Handover Analysis

**Figure 3:**
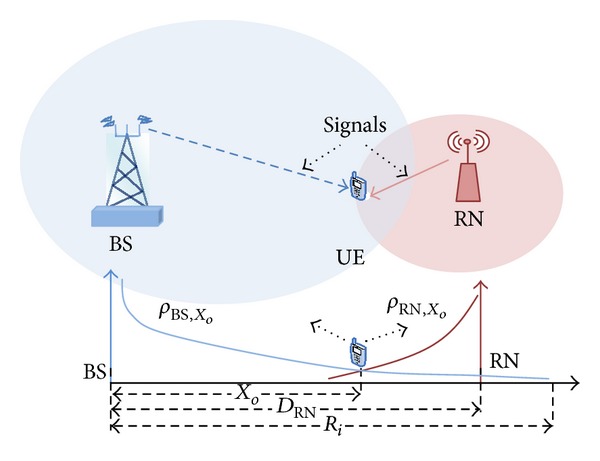
Hand over procedure.

In multihop relaying the handover process becomes more important and a difficult importance within any cellular network. It is necessary to guarantee that it can be implemented reliably and without disruption to any wireless service. A fixed relay-based LTE-architecture that fits perfectly to improve the SINR at the cell boundary, thereby increasing capacity, can possibly increase the number of accepted users. However, the relay node and base station are located at a certain distance from each other, in which the SINR at UE from the relay link is equal to the SINR for direct link, where the user is in a handover case [17]. Therefore the distance from the BS to the said location is *X*_*o*_ which is a known handover distance as shown in Figure 3.

To evaluate the *X*_*o*_, first evaluating the received signals from both BS and RN at UE in *X*_*o*_ location putting the distance from relay location (*D*_RN_) to *X*_*o*_ is *D*_RN_ − *X*_*o*_. Thus, the received signal from the BS at the UE in *X*_*o*_ point can be expressed as

(15) $$\begin{array}{c} Y_{i,X_{o}}=\sqrt{P_{i}}H_{i,X_{o}}X_{i,X_{o}}+\sqrt{P_{\text{RN}}}H_{\text{RN},X_{o}}X_{\text{RN},X_{o}}+N_{X_{o}}. \end{array}$$

SINR at *X*_*o*_ through direct link is

(16) $$\begin{array}{c} \rho_{i,X_{o}}=\frac{P_{i}{\left|H_{i,X_{o}}\right|}^{2}}{P_{\text{RN}}{\left|H_{\text{RN},X_{o}}\right|}^{2}}, \end{array}$$

(17) $$\begin{array}{c} \rho_{i,X_{o}}=\frac{P_{i}LX_{o}^{-\alpha }}{P_{\text{RN}}L_{r}{\left(D_{\text{RN}}-X_{o}\right)}^{-\alpha }}, \end{array}$$

where *L*_*r*_ = *G*_*r*_ *G*_*t*_(*λ*/4*π*)^2^ is the relay node characteristic and *G*_*r*_ *G*_*t*_ are antenna gains of transmitter and receiver, respectively. The received signal from the relay link can be expressed as

(18) $$\begin{array}{c} Y_{\text{RN},X_{o}}=\sqrt{P_{\text{RN}}}H_{\text{RN},X_{o}}X_{\text{RN},X_{o}}+\sqrt{P_{i}}H_{i,X_{o}}X_{i,X_{o}}+N_{X_{o}}, \end{array}$$

(19) $$\begin{array}{c} \rho_{\text{RN},X_{o}}=\frac{P_{\text{RN}}{\left|H_{\text{RN},X_{o}}\right|}^{2}}{P_{i}{\left|H_{i,X_{o}}\right|}^{2}}, \end{array}$$

(20) $$\begin{array}{c} \rho_{\text{RN},X_{o}}=\frac{P_{\text{RN}}L_{r}{\left(D_{\text{RN}}-X_{o}\right)}^{-\alpha }}{P_{i}LX_{o}^{-\alpha }}. \end{array}$$

At the *X*_*o*_ the received signal at UE from BS is equal to the signal at UE rather than RN. This location is known as the handover point, as shown in Figure 3. Using (17) and (20) to evaluate *X*_*o*_ through equal received SINR from both BS and RN within handover location is as follows:

(21) PiLXo−αPRNLr(DRN−Xo)−α=PRNLr(DRN−Xo)−αPiLXo−α,(PBSXo−α)(PBSXo−α)  =(PRN(DRN−Xo)−α)(PRN(DRN−Xo)−α),PRN−1/αLrDRN=Xo(LPi−1/α+LrPRN−1/α),Xo=DRN((LrPRN/LPi)1/α+1).

Based on (21), *X*_*o*_ is a distance dependent on the relay location, the node characteristics, and path loss exponent.

The equations of relay coverage are calculated, which are determined by the locations of UE and presented in Figure 2. Therefore the received signals at UE in locations 2 and 3 can be represented as

(22) $$\begin{array}{c} Y_{\text{RN},2}=\sqrt{P_{\text{RN}}}B_{\text{RN},2}X_{\text{RN},2}+\sqrt{P_{i}}B_{i,2}X_{i,2}+N_{2},\\ Y_{\text{RN},3}=\sqrt{P_{\text{RN}}}B_{\text{RN},3}X_{\text{RN},3}+\sqrt{P_{i}}B_{i,3}X_{i,3}+N_{3}. \end{array}$$

The SINR for locations 2 and 3 in Figure 2 is

(23) $$\begin{array}{c} \rho_{\text{RN},2}=\frac{L_{r}P_{\text{RN}}{\left|B_{\text{RN},2}\right|}^{2}}{P_{i}L_{i}{\left|B_{i,2}\right|}^{2}},\;\;\rho_{\text{RN},3}=\frac{P_{\text{RN}}{\left|B_{\text{RN},3}\right|}^{2}}{P_{i}{\left|B_{i,3}\right|}^{2}},\\ \rho_{\text{RN},2}=\frac{P_{\text{RN}}L_{r}{\left(D_{\text{RN}}-D_{i}\right)}^{-\alpha }}{P_{i}L{D_{i}}^{-\alpha }},\\ \rho_{\text{RN},3}=\frac{P_{\text{RN}}L_{r}{\left(D_{i}-D_{\text{RN}}\right)}^{-\alpha }}{P_{i}LD_{i}^{-\alpha }}. \end{array}$$

Then the spectral efficiency of relaying system has been divided into four regions to explain the coverage distribution over cell edges according to RN location. Therefore the analytical expression of the rising and decline of spectral efficiency level, taking on the account of the saturation region, is explained with the following equations:

(24) CRN,2 =min⁡{BWeff log2(1+PRNLr(DRN−Di)−αPiLρeffDi−α),CRmax⁡},CRN,3 =min⁡{CRmax⁡,BWeff log2(1+PRNLr(Di−DRN)−αPiLρeffDi−α)}.

*C*_*R*max⁡_ is the maximum spectral efficiency of relay that depends on the transmitted power relay and antenna infrastructure. Where the domains of *D*_*i*_ are *X*_*o*_ < *D*_*i*_ < *X*_*s*1_, *X*_*s*2_ < *D*_*i*_ < *R* for *C*_RN,2_, *C*_RN,3_, respectively.

### 2.3. Optima Relay Location for LTE-A Cellular Networks

In this section, the issue of the optimum placement of the relay node deployment in a dual-hop network over LTE-A cellular networks will be addressed as well as the maximum throughput and limited interference between all in-band and out-band stations.

The optimum location of relay at the cell edge between two locations of the user when *D*_*i*_ = *X*_*o*_ and *D*_*i*_ = *R* is used as the relay capacity equations can be obtained through the following:

(25) $$\begin{array}{c} \frac{{\left(D_{\text{RN}}-X_{o}\right)}^{-\alpha }}{X_{o}^{-\alpha }}=\frac{{\left(R-D_{\text{RN}}\right)}^{-\alpha }}{R^{-\alpha }},\\ X_{o}\left(R-D_{\text{RN}}\right)=R\left(D_{\text{RN}}-X_{o}\right). \end{array}$$

Substituting (21) in (25), we obtain

(26) $$\begin{array}{c} D_{\text{RN}}=R\left(1-{\left(\frac{L_{r}P_{\text{RN}}}{LP_{i}}\right)}^{1/\alpha }\right). \end{array}$$

From (26) the relay location depends on the cell radius, the properties of the relay node and the BS, and *α*. This equation limits the relay location between handover point and the boundaries of cell.

## 3. Group Mobility Analysis

In this section, a mobility model is proposed, where all UEs in vehicle are moving across of cell at different velocities. According to the derived formulas of instantaneous SNR at direct and relay links in [1], the group mobility for MR and user has been derived in order to evaluate the system performance and reducing the transmitted power of MR. Therefore both UEs and MR are moving as group mobility across BS.

Typically, the instantaneous SNR changes according to environment of channel, such as the distance between the transmitter and receiver, and fading state of the channel.

The user in vehicle receives two signals: via direct link and from MR via relay link; therefore the combined SNR at UE is

(27) $$\begin{array}{c} \rho_{\text{UE}}^{\text{MRC}}=\rho_{\text{UE}}^{\text{DL}}+\rho_{\text{UE}}^{\text{MR}}, \end{array}$$

where the *ρ*_UE_^DL^, *ρ*_UE_^MR^ is SNR at UE via direct and relay links, while *ρ*_UE_^MRC^ is the combination of SNR at UE via both the direct and relay links.

By inserting (4) the result is

(28) $$\begin{array}{c} \rho_{\text{UE}}^{\text{DL}}=\frac{P_{i}L{\left(d_{\text{DL}}\right)}^{-\alpha }}{N_{o}},\\ \rho_{\text{UE}}^{\text{MR}}=\frac{P_{i}P_{\text{MR}}L{\left(d_{\text{MR}}\right)}^{-\alpha }{\left|H_{\mathrm{AL}}\right|}^{2}}{\left[P_{i}L{\left(d_{\text{MR}}\right)}^{-\alpha }+2P_{\text{MR}}{\left|H_{\mathrm{AL}}\right|}^{2}+N_{o}\right]N_{o}}. \end{array}$$

**Figure 4:**
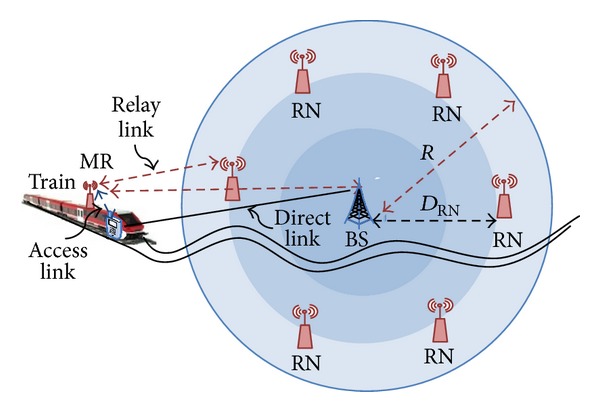
Vehicle travelling across a one cell size.

*d*_MR_ is the distance between the BS and MR, *P*_MR_ is the transmitted power by MR, and *H*_*AL*⁡_ is the channel gain of access link between the user and MR inside vehicle as shown in Figure 4.

Intuitively the distance is function of velocity and time so

(29) $$\begin{array}{c} \rho_{\text{UE}}^{\text{DL}}=\frac{P_{i}L{\left(v_{\text{UE}}T_{\text{UE}}\right)}^{-\alpha }}{N_{o}}, \end{array}$$

(30) $$\begin{array}{c} \rho_{\text{UE}}^{\text{MR}}=\frac{P_{i}P_{\text{MR}}L{\left(v_{\text{MR}}T_{\text{MR}}\right)}^{-\alpha }{\left|H_{\mathrm{AL}}\right|}^{2}}{\left[P_{i}L{\left(v_{\text{MR}}T_{\text{MR}}\right)}^{-\alpha }+2P_{\text{MR}}{\left|H_{\mathrm{AL}}\right|}^{2}+N_{o}\right]N_{o}}, \end{array}$$

(31) $$\begin{array}{c} {C_{\text{UE}}^{\text{MRC}}}_{}=\text{B}{\text{W}}_{\text{eff}}\;\text{lo}{\text{g}}_{2}\left(1+\frac{\rho_{\text{UE}}^{\text{MRC}}}{\rho_{\text{eff}}}\right). \end{array}$$

### 3.1. Balancing Power Algorithm (BPA) of MR

The SNR at (29) and (30) depends on the transmitted power and path loss between both transmitter and receiver, so the proposed BPA is balanced and controlled on the transmitted power of MR over cell radius to achieve the required SNR and throughput at the users with mitigation of the consumption in transmitted relay power.

Typically the coverage distribution close to BS is better than boundaries and therefore does not require consumption additional power when the vehicle (i.e., train, bus, metro) passes near BS where there is a good SNR. The proposed BPA depends on this idea as explained in Figure 4.

Two constraints are proposed in algorithm:

(32) minimize PRN subject 0<PMR≤Pmax⁡ρth<ρUEMR≤ρmax⁡ρUEDL≤ρth,

where *ρ*_th_, *ρ*_max⁡_ is the threshold and maximum required SNR at the UE.

Inputs for Algorithm 1 are that *ρ*_th_, *ρ*_max⁡_ is the threshold and maximum required SNR at the UE. *P*_max⁡_, *P*_min⁡_, is maximum and minimum level of power transmitted by MR. *Q* is the number of users at the vehicle, while *V*_RN_ is velocity of vehicle. *Q*_MR-UE_ and *Q*_BS-UE_ are the number of users which are attached with MR and BS, respectively. *P*_MR_ is the power transmitted by MR. *ρ*_UE,*q*_^Direct^, *ρ*_MR_, and *ρ*_UE,*q*_^Access^ is the SNR at direct, MR, and access links, respectively.

The main body of balancing algorithm is described line by line as the following steps in Algorithm 1.

*Line 1*. Beginning of algorithm.

*Line 2*. Selection of the number of users which are attached with MR or BS. 

*Line 3*. Calculating the SNRs at the direct, relay, and access links for each user. 

*Line 4*. Comparison between the SNRs at direct and access links and then determination of the better link in order to enable it and disable the other. 

*Line 5*. In case *ρ*_UE,*q*_^Direct^ is better than the user attached directly with BS, the number of users that are attached with BS will increase by one. 

*Line 6*. Enable the transmitted power of relay equal minimum chosen value. 

*Line 7–12*. Algorithm proposed a second comparison between the SNR at relay link and required threshold SNR of system. If *ρ*_MR_ ≥ *ρ*_th_ the number of users that are attached with MR will increase by one and enable the transmitted power of MR equal minimum chosen value. Otherwise enable the transmitted power of MR equal maximum chosen value. 

*Line 13-14*. In this line there is counter to calculate the instantaneous value of *ρ*_MR_ at each user according to the distance between the MR and BS. Typically when the MR close to BS the *ρ*_MR_ is high, this link will degrade when MR is away from BS. 

*Line 15–18*. Comparison between the *ρ*_MR_ and maximum chosen SNR in order to balance and save the transmitted power by MR and reducing the power consumption of MR. This step limits the transmitted power according to quality of received signal strength at the users in vehicle. 

*Line 19–22*. Closed if and for statements.

## 4. Simulation Setup

The signal strength in the service area must be measured to design a more accurate coverage of modern LTE networks. The propagation of a radio wave is a complicated and less predictable process if the transmitter and receiver properties are considered in channel environment calculations. The process is governed by reflection, diffraction, and scattering; the intensities of which vary under different environments at different instances.

The ATDI simulator, used to approve the mathematical model for optimum relay placement. The propagation model for this simulator between the nodes can be expressed as the following equation:

(33) $$\begin{array}{c} P_{r}=P_{t}+G_{t}+G_{r}-L_{\text{prop}}-L_{t}-L_{\text{re}}\;\left[\text{dB}\right], \end{array}$$

where *P*_*t*_ indicates the power at the transmitter and *P*_*r*_ is the power at the receiver; *G*_*t*_ and *G*_*r*_ are the transmitter and receiver antenna gains, respectively; *L*_*t*_ and *L*_re_ express the feeder losses; and *L*_prop_ is the total propagation loss [18], formulated as

(34) $$\begin{array}{c} L_{\text{prop}}=L_{\text{fsd}}+L_{d}+L_{\text{sp}}+L_{\text{gas}}+L_{\text{rain}}+L_{\text{clut}}, \end{array}$$

where *L*_fsd_ is the free space distance loss, *L*_*d*_ is the diffraction loss, *L*_sp_ is the sub path loss, *L*_gas_ is the attenuation caused by atmospheric gas, *L*_rain_ is the attenuation caused by hydrometeor scatter, and *L*_clut_ is the cutter attenuation.

**Figure 5:**
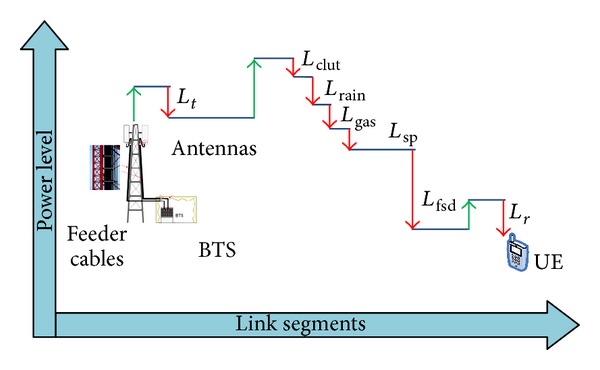
Link budget scheme.

This equation describes the link budget. A link budget describes the extent to which the transmitted signal weakens in the link before it is received by the receiver. The link budget depends on all the gains and losses in the path, which is facing the transmitted signal to reach the receiver. A link is created by three related communication entities: transmitter, receiver, and a channel (medium) between them. The medium introduces losses caused by suction in the received power, as shown in Figure 5.

The SINR at the user equipment over the simulation test can be explained by using the following equation:

(35) $$\begin{array}{c} \text{SIN}{\text{R}}_{\text{sim}}=\frac{P_{r}}{N_{o}+\sum_{j=1}^{j=N}P_{rj}}, \end{array}$$

where the SINR_sim_ is the received SINR by the user and calculated by the simulator; *P*_*rj*_ is the received signal from the neighbouring cell; and *j* = {1,…, *N*}, where *N* is the number of neighbouring cells. For simplicity, we suggested the use of the first tier (six cells around the centralized cell) in planning for an urban area, with *N*_*o*_ as the background noise at the receiver:

(36) $$\begin{array}{c} \text{SIN}{\text{R}}_{\text{sim}}=\frac{P_{t}G_{t}G_{r}/L_{\text{prop}}L_{t}L_{\text{re}}}{N_{o}+\sum_{j=1}^{j=N}P_{j}G_{t,j}G_{r}/L_{\text{prop},j}L_{j}L_{\text{re}}}, \end{array}$$

where *L*_*t*_, *L*_*j*_, and *L*_re_ are the feeder loss for senders (central BS and the surrounding BS_*j*_) and destination:

(37) $$\begin{array}{c} C_{\text{sim}}=0.5\;\text{lo}{\text{g}}_{2}\left(1+\text{SIN}{\text{R}}_{\text{sim}}\right),\\ C_{\text{sim}}=0.5\;\text{lo}{\text{g}}_{2}\left(1+\frac{P_{t}G_{t}G_{r}/L_{\text{prop}}L_{t}L_{\text{re}}}{N_{o}+\sum_{j=1}^{j=N}P_{j}G_{t,j}G_{r}/L_{\text{prop},j}L_{j}L_{\text{re}}}\right). \end{array}$$

## 5. Results and Discussion

In this section, the numerical results for the proposed mathematical model are explained and compared with results using the ATDI simulator, which uses a real digital cartographic representation of an urban area.

**Figure 6:**
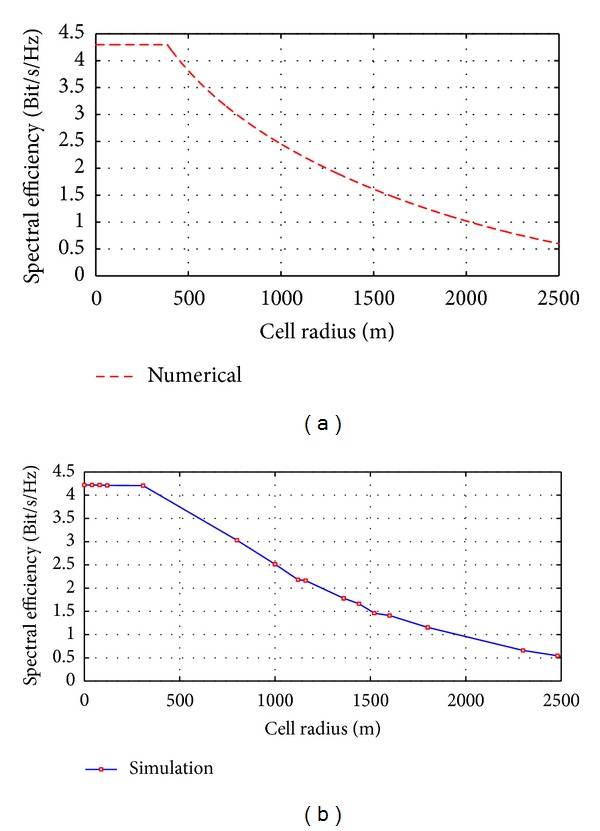
Spectral efficiency versus the cell radius: (a) numerical results and (b) ATDI simulation results.

Figure 6(a) explains the downlink spectral efficiency versus cell radius without relaying scenario with consideration of the throughput saturation distance near station placement which is estimated here 306 m from BS as shown in Figure 6(a).  Figure 6(b) shows the simulation analysis which considered the interferences for the first tier (six cells around the main cell) by using ATDI simulator.

These figures show the system performance degradation at the cell edge region when the user is away from BS.

**Figure 7:**
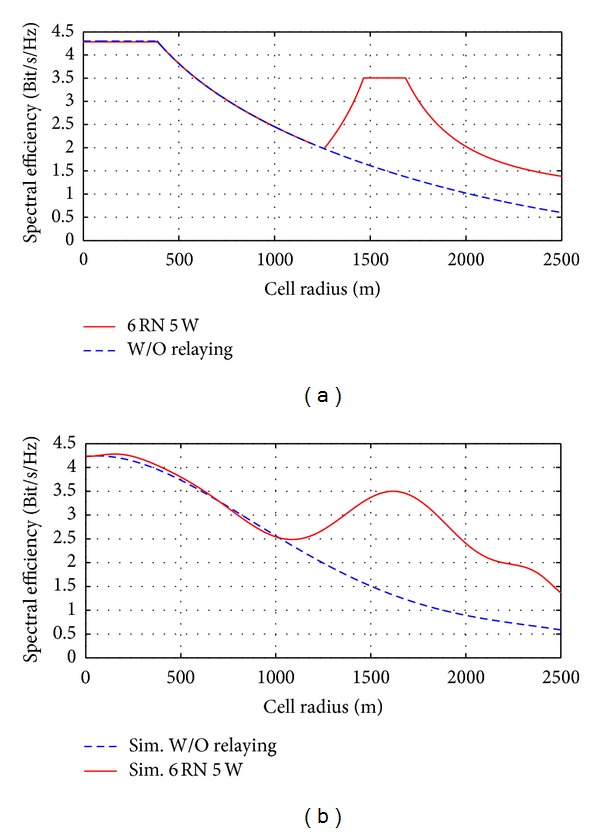
Spectral efficiency enhancement by deploying six RN at optimal location: (a) numerical and (b) simulation.

Figure 7 displays the numerical and simulation curves are the results of the proposed mathematical model for optimal location (1660 m from the BS) by using (26). This model aims to improve the capacity and signal strength at the cell edge while mitigating interference between the stations. According to the proposed model the spectral efficiency at cell edge is improved from 0.6 bps/Hz to 1.45 bps/Hz for the proposed optimal location.  Figure 7(a) describes the enhancement in spectral efficiency which is obtained by numerical analysis, while Figure 7(b) represents the simulation results by using ATDI simulator.

The difference between numerical and simulation results is due to the simulator since the ATDI simulator deals with a real digital cartographic that contains several types of clutters for path loss conditions based on (34).

**Figure 8:**
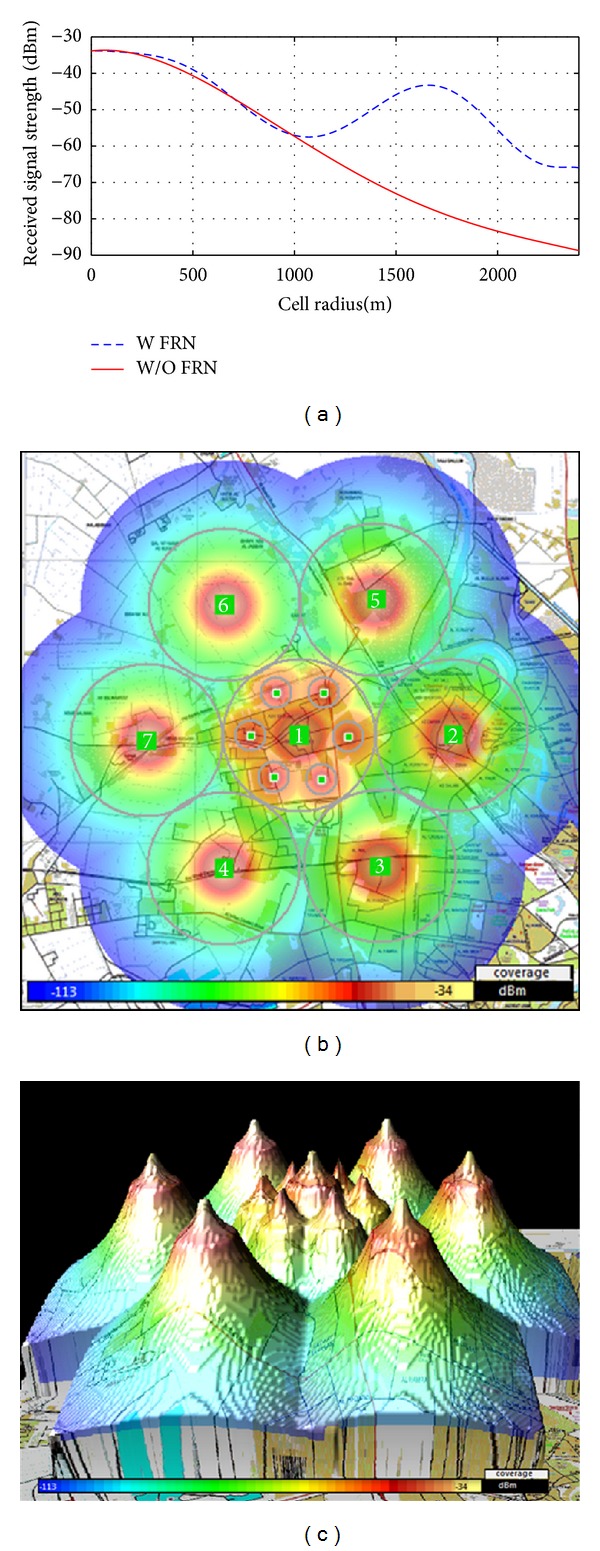
Received signal strength versus the distance with a real digital cartographic of an urban city for 6RN 5W, (a) received signal strength versus the distance, (b) 2-dimansion chromatic scheme of coverage area distribution, and (c) 3-dimansion chromatic scheme of coverage area distribution.

Figure 8 demonstrates the numerical and simulation results in received signal strength at the UE for proposed optimal location.  Figure 8(a) shows the enhancement of the received signal strength after deploying six RN around BS in proposed location which is 40% for cell edge region according to simulated parameters as indicated in Table 1.

Figures 8(b) and 8(c) show chromatic scheme of coverage area distribution for 2-dimansion and 3-dimansion schemes, respectively, after deploying six relays each one 5 watts installed at optimal location (1660 m). These figures explain the coverage extension along with mitigating the interference between them. It should be noted here that ATDI simulator is based on a real digital cartographic representation of an urban area that is approximately 176.7 km^2^.

**Figure 9:**
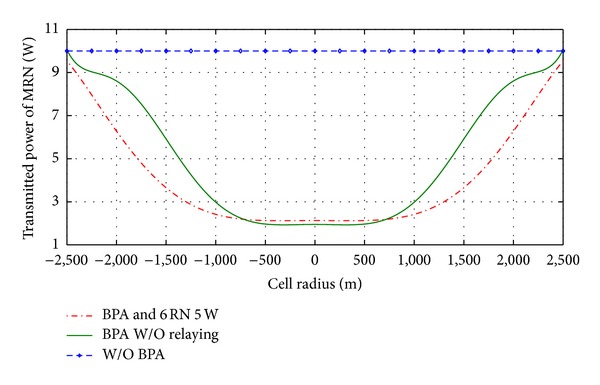
Reducing power consumption by using BPA.

Figure 9 shows the reducing in transmitted power of MR together with providing the required throughput and SNR at each user inside vehicle. The saving in transmitted power of relay is approximately 60% from transmitted power in MR after proposed BPA and 75% saving in transmitted power with deploying six RN at proposed optimal relay location. BPA balanced the power along changing the distance between the vehicle and BS in order to insure maximum SNR at users and saving an extra power from MR as shown in Figure 9.

The number of active user is increased by installing MR node above the vehicle moreover the throughput for each user enhanced too compared with throughput at user in vehicle did not include MR as shown in Figure 9. This enhancement in throughput could be more with deploying six RN around BS at optimal proposed location as shown in Figure 9.

## 6. Conclusion

In this paper, we discussed two issues: the first is deriving the optimal relay placement in order to increase the capacity and coverage extension at LTE-A cellular network. This deriving of optimal RN placement was dependent on mathematical analysis and taken on account mitigating the interference among the nodes. The second is proposed BPA which saved the extra transmitted power of MR along with providing the required SNR and throughput at each user inside vehicle.

**Figure 10:**
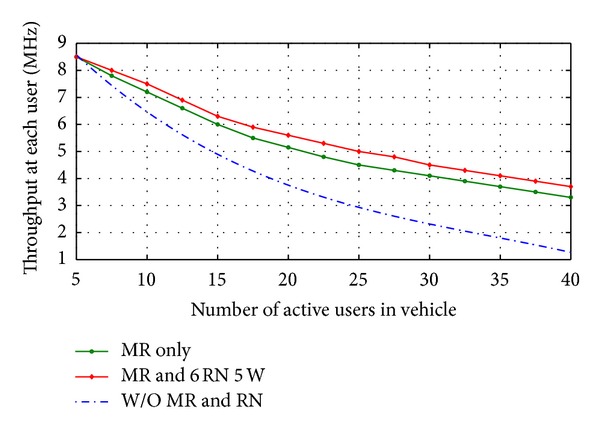
Throughput enhancement at users inside vehicle.

The numerical results together with simulation results indicated that there is 40% enhancement of capacity and received signal at users in cell edge region (Figure 10). Moreover there is 75% saving in transmitted power of MR achieved by proposing BPA with enhancing throughput for passengers.

## Figures and Tables

**Algorithm 1:**
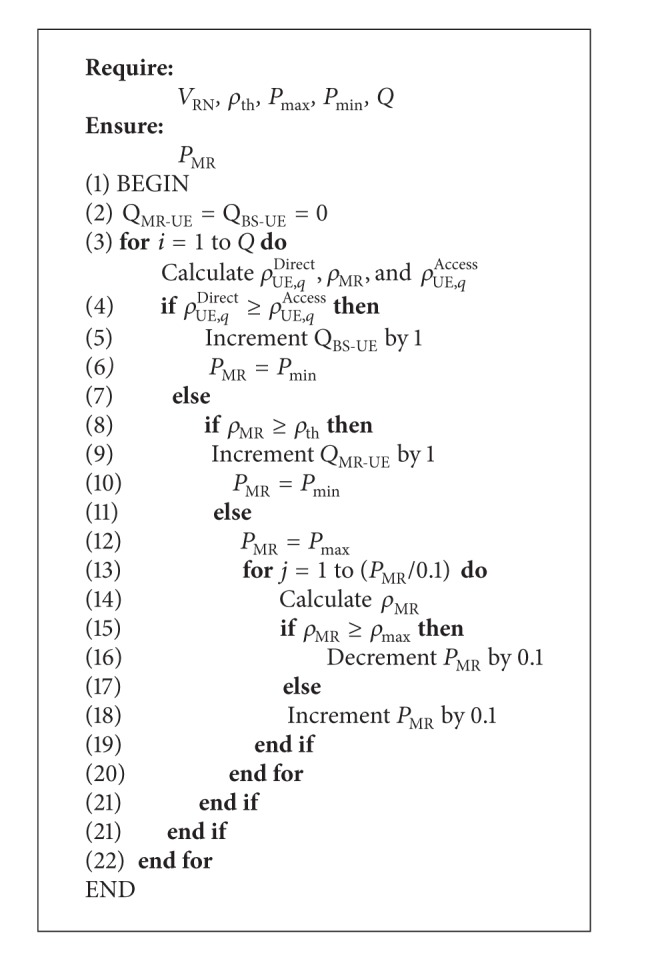
Balancing power algorithm of reducing and balancing of transmitted power consumption at MR.

**Table 1:** Simulation parameters.

Carrier frequency GHz	2
Bandwidth	1.4 MHz
Number of BS	7
Antenna height of BS	25 (m)
Antenna gain	17 dBi
Type of antenna	Omidirectional
Transmitted power of BS	40 W
Radius of cell	2500 m
Antenna height of RN	25 m
Antenna gain of RN	5 dBi
Transmitted power of RN	5 Watt
Number of UE	1
Antenna height of UE	1.5 m
Antenna gain	0 dBm
Coverage threshold	−30 dBm
